# Time course of neurological deficits after surgery for primary brain tumours

**DOI:** 10.1007/s00701-020-04425-3

**Published:** 2020-07-02

**Authors:** Maria Zetterling, Kristin Elf, Robert Semnic, Francesco Latini, Elisabeth Ronne Engström

**Affiliations:** 1 Department of Neuroscience, Neurosurgery, University Hospital, Uppsala University, S-751 85 Uppsala, Sweden; 2 Department of Neuroscience, Clinical Neurophysiology, University Hospital, Uppsala University, S-751 85 Uppsala, Sweden; 3 Department of Surgical Sciences, Radiology, Uppsala University, S-751 85 Uppsala, Sweden

**Keywords:** Brain tumour surgery, Postoperative neurological deficit, Complications, Time course

## Abstract

**Background:**

The postoperative course after surgery for primary brain tumours can be difficult to predict. We examined the time course of postoperative neurological deficits and analysed possible predisposing factors.

**Method:**

Hundred adults with a radiological suspicion of low- or high-grade glioma were prospectively included and the postoperative course analysed. Possible predictors of postoperative neurological deterioration were evaluated.

**Results:**

New postoperative neurologic deficits occurred in 37% of the patients, and in 4%, there were worsening of a preoperative deficit. In 78%, the deficits occurred directly after surgery. The probable cause of deterioration was EEG-verified seizures in 7, ischemic lesion in 5 and both in 1, resection of eloquent tissue in 6, resection close to eloquent tissue including SMA in 11 and postoperative haematoma in 1 patient. Seizures were the main cause of delayed neurological deterioration. Two-thirds of patients with postoperative deterioration showed complete regression of the deficits, and in 6% of all patients, there was a slight disturbance of the function after 3 months. Remaining deficits were found in 6% and only in patients with preoperative neurological deficits and high-grade tumours with mainly eloquent locations. Eloquent tumour location was a predictor of postoperative neurological deterioration and preoperative neurological deficits of remaining deficits.

**Conclusions:**

Postoperative neurological deficits occurred in 41% and remained in 6% of patients. Remaining deficits were found in patients with preoperative neurological deficits and high-grade tumours with mainly eloquent locations. Eloquent tumour location was a predictor of neurological deterioration and preoperative neurological deficits of remaining deficits.

**Electronic supplementary material:**

The online version of this article (10.1007/s00701-020-04425-3) contains supplementary material, which is available to authorized users.

## Introduction

After surgery for primary brain tumours, it is not uncommon with a deterioration of the neurological function [6, 7, 21, 15]. In some cases, postoperative neurological deterioration is expected due to either perioperative ischemic injury or surgery in eloquent areas with corresponding deficits or when a supplementary motor area (SMA) syndrome occurs after surgery in the premotor cortex [17]. However, the reason for the postoperative neurological decline is not always clear, and often, it is difficult to predict the course of the deteriorated function. In the preoperative information to the patient, it is desirable to give more precise information regarding postoperative outcome. The primary aim of this study was to analyse the occurrence and time course of postoperative neurological deficits and the secondary aim to find possible predisposing factors.

## Methods and materials

### Patients

One hundred patients, with a presumed glioma (WHO grades II–IV) planned for surgery at the Department of Neurosurgery, Uppsala University Hospital during the period 22 August 2016 to 7 December 2017, were prospectively included. There were 60 men and 40 women with a mean age of 53.5 ± 16.2 years.

Preoperative neurological deficits were evaluated with clinical examination by a specialist in neurosurgery and documented in the medical record the day before surgery. The motor deficit was scored according to our clinical scale used for pre- and postoperative evaluation: no motor deficit – discrete motor deficit, pronounced motor deficit or complete motor deficit. Cognitive deficit was defined as the presence of confusion, disorientation, personality change or memory disturbances judged by clinical examination or medical reports.

Eloquent tumour location was assessed according to Chang et al. [5]. The presumed eloquent areas included sensorimotor strip (precentral and postcentral gyri), dominant hemisphere perisilvian language areas (superior temporal, inferior frontal and inferior parietal areas), basal ganglia/internal capsule, thalamus and calcarine visual cortex.

#### Surgery and postoperative care

The surgical procedure is described in detail earlier [13]. Briefly, tumour resection was done through craniotomy using microsurgery guided by neuronavigation and intraoperative ultrasound. Intraoperative neurophysiological monitoring of motor function was performed if the tumour was located in close connection to eloquent cortical or subcortical areas. In an awake surgery, speech function and visual fields were monitored. 5-Amnolevulinic acid (5-ALA) (Gliolan, Medac Pharma, Varberg, Sweden) was used in 21 patients with presumed high-grade (contrast enhancing) tumours if total resection was the goal of surgery. After surgery, the patient was awakened in the operating theatre and brought to the postoperative neurointermediate ward, and EEG- and video monitoring was initiated [13]. A neurologic examination was performed by the responsible surgeon. The level of consciousness according to Reaction Level Scale 85 (RLS85) [31] and the presence and grade of postoperative neurological deficits were then monitored according to our clinical postoperative protocol by specially trained nurses. According to his protocol, RLS85 is checked every 30 min and neurological status (motor deficits) every 60 min for the first 6 h, RLS85 and neurological status every 60 min for 6–12 h postoperatively and every 120 min 12–24 h after surgery. After 24 h, the monitoring is prolonged if indicated in selected cases. An acute CT scanning was performed in any case of postoperative deterioration or new neurological deficits. In uncomplicated cases, postoperative monitoring continued for 24 h in the neurointermediate ward, and the patient was then discharged to the general ward. If there was a complicated postoperative course, for example, with seizures or new neurological deficits, postoperative monitoring in the neurointermediate ward continued until the patient was considered stable.

Patients were followed up and the neurological status checked in the outpatient clinic 3 months postoperatively. In some patients with high-grade tumours undergoing oncological treatment, the neurological status was evaluated by the responsible doctor. A complete regression was defined as no visible deficits left, and the performances of the patient were unchanged compared with those of before surgery. An almost complete regression was defined as a there was a slight remnant of the deficits, but the patient was not impaired by it in daily life and it was not clearly visible for the examiner. Remaining deficits were defined as the deficits were still there (but might had improved) at the 3 months following up.

#### Radiology

Postoperative magnetic resonance imaging (MRI) was performed within 48 h after surgery. In contrast enhancing (high-grade) tumours, contrast enhancement on T1-weighted turbo spin echo sequences and in non-contrast enhancing tumours, high signal intensity on T2 fluid-attenuated inversion recovery (FLAIR) sequences was considered a tumour tissue.

Postoperative ischemic lesions were evaluated on DWI with B 1000 value and corresponding ADC map. The total volume of ischemic lesion (in cm^3^) was calculated using the Vue PACs software (Picture Archiving Communication System, v11.1.4) and its semi-automated lesion management application (livewire algorithm) [20]. The software is supported by an algorithm that uses an active contour model in order to evolve and segment the lesions. In defining the volume of the surface voxels, a clear difference in pixel contrast (black/white) assisted the operator, increasing the ability to better adapt or correct the ischemic contour line even where it was less defined. To investigate the correlation between ischemic lesions and white matter tracts, FLAIR and DWI sequences were normalized into MNI space using the built-in software of DSI studio (DSI Studio, http://dsi-studio.labsolver.org/download-images). The ischemic areas were defined as new regions of interests (ROIs) on patient-specific sequences and reconstructed into the HCP-1021 template. A group average template was constructed from a total of 1021 subjects enrolled by the Human Connectome Project (the WU-Minn HCP consortium which is an institutional, review board–approved, NIH-funded project led by Washington University, University of Minnesota and Oxford University) [35]. A multishell diffusion scheme was used, and the *b* values were 990, 1985 and 2980 s/mm^2^. The number of diffusion sampling directions was 90, 90 and 90, respectively. The in-plane resolution was 1.25 mm. The slice thickness was 1.25 mm. The diffusion data were reconstructed into MNI space using q-space diffeomorphic reconstruction [41] to obtain the spin distribution function [42]. A diffusion sampling length ratio of 2.5 was used, and the output resolution was 1 mm. The restricted diffusion was quantified using restricted diffusion imaging [40]. Major projection, commissural and association white matter pathways were reconstructed within the HCP-1021 template following the anatomical criteria already published with the Brain Grid DTT reference atlas [19] and matched with ROIs defining the ischemic areas. The method’s workflow is visually described in Supplementary Fig. 1. The white matter structures impinged by the ischemic lesions are displayed in Table 4.

### Statistics

Comparisons between groups were made with Mann-Whitney *U* test for continuous and categorical variables and Fischer exact two-tailed test for proportions. Possible predictors of postoperative neurological deterioration were evaluated in a simple regression analysis. Factors with a *p* value < 0.1 were chosen to be tested in the multiple regression analysis. A *p* value < 0.05 was considered statistically significant. Statistica, version 13.2 (StatSoft, Inc. Tulsa, OK, USA), was used for statistical calculations.

### Ethics

The study was approved by the institutional ethics review board (2016/112). Informed consent was obtained prior to participation.

## Results

### Tumour locations, diagnosis and tumour volumes

Tumour locations and tumour diagnosis are presented in Table 1. The most common tumour location was frontal, *n* = 33 (33%) followed by temporal, *n* = 27 (27%). Forty patients had a right-sided tumour, 52 patients left-sided, 6 patients bilateral and 2 patients had midline tumours. Thirty-nine patients harboured tumours in presumed eloquent areas (Table 1) and two patients in the SMA. Intraoperative neurophysiological monitoring of motor function was used in 25 patients which was combined with awake surgery and monitoring of speech functions in 10 of these patients.

**Table 1:** Tumour locations and diagnosis in 100 patient

	*n* = %	Eloquent area (*n*)
Tumour location		
Frontal	33	12
Temporal	27	8
Parietal	4	3
Occipital	2	2
Insular*	12	11
Frontal + corpus callosum/ gyrus cinguli	8	
Frontal-parietal-temporal	1	1
Temporal-occipital	4	1
Parietal-temporal	4	
Parietal-occipital	4	1
Midline	1	
Tumour diagnosis		
WHO grade IV	*50*	
Glioblastoma	48	
Gliosarcoma	2	
WHO grade III	*19*	
Anaplastic Astrocytoma	11	
Anaplastic Oligodendroglioma	6	
Ependymoma III	1	
Anaplastic pleomorft xantoastroctoma	1	
WHO grade II	*24*	
Astrocytoma	9	
Oligodendroglioma	13	
Ependymoma	1	
Not classified	1	
WHO grade I	*1*	
Pilocytic astrocytoma	1	
Metastasis	*2*	
Adenocarcinoma	1	
Gastric carcinoma	1	
Other	*4*	
B-cell lymphoma	1	
Unclassified	3	

Numbers in italics are the total numbers in every subgroup

*Fronto-insular, *n* = 2; temp-insular, *n* = 1; fronto-temporal-insular, *n* = 8; fronto-temporal-insular + central, *n* = 1. The presumed eloquent areas were sensorimotor strip (precentral and postcentral gyri), dominant hemisphere perisilvian language areas (superior temporal, inferior frontal and inferior parietal areas), basal ganglia/internal capsule, thalamus and calcarine visual cortex

In 21 of the patients with tumours, according to Chang [5], in eloquent areas, neurophysiological monitoring was used. However, 18 of the patients with eloquent tumour location went through surgery without neurophysiological monitoring. In 8 of these patients, a part of the tumour extended into the basal ganglia, and the intention was not to resect this portion. Ten of the patients showed preoperative neurological deficits, and radical surgery was not planned (*n* = 7) or the deficits were already maximal and considered not to be worsened by surgery (3 patients with hemianopsia).

In 5 patients with tumour location that was not considered eloquent according to Chang, neurophysiological monitoring of the motor functions was used due to close connection to subcortical motor tracts (3 patients with tumours in the parietal area) or cortical motor areas and subcortical motor tracts (2 patients with tumours in the SMA).

The most common diagnosis was high-grade glioma, found in 69 patients (69 %) followed by low-grade glioma (WHO grade II) in 24 patients (24%). Median (IQR) preoperative tumour volume was 32.4 (11.2–74.5) cm^3^ and resection grade was 96.5 (72–100) %.

### Pre- and postoperative neurological deficits

**Fig. 1:**
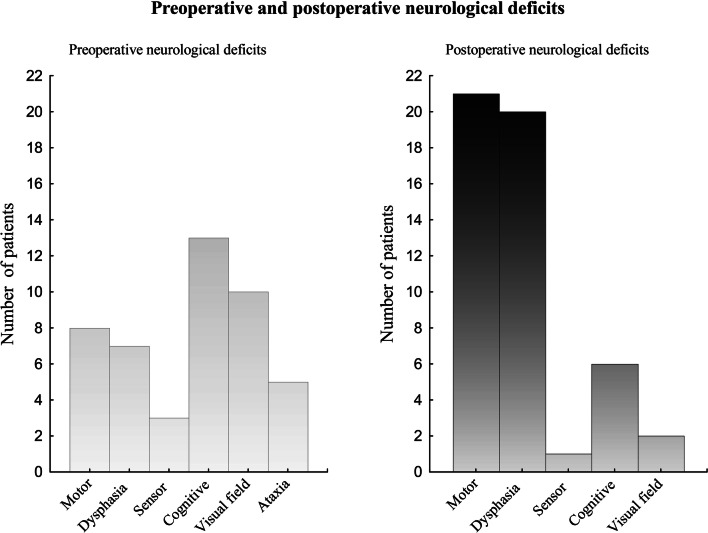
Pre- and postoperative neurological deficits. Before surgery (left), 40 patients displayed neurological deficits of whom six patients had two deficits. After surgery (right), 37 patients showed new neurological deficits, and four patients exhibited worsening of a preoperative existing neurological deficits. In 13 of these patients, there were two deficits

Pre- and postoperative neurological deficits are shown in Fig. 1. Preoperative neurological deficits were present in 40 patients. Cognitive deficit was the most common (13%), followed by visual field (10%) and motor deficit (8%). New postoperative neurologic deficits were found in 37% of patients. In addition, 4% of patients exhibited worsening of a preoperative existing neurological deficit postoperatively. Most commonly, motor dysfunction occurred (21 patients) and 20 patients showed dysphasia.

Among the 25 patients who went through intraoperative neurophysiological monitoring, 18 patients developed new postoperative deficit. Six of these patients went through awake surgery, and in 12 patients, intraoperative monitoring of motor function was used. In 2 patients with neurologic deterioration after awake surgery, there were intraoperative fluctuations of speech functions which made intraoperative speech evaluation difficult and one patient showed dysarthria due to motor impairment of the tongue (with intact motor signals). In the remaining 3 patients, there were only stimulation-induced speech disturbances which is an expected finding at awake surgery. Among the 12 patients with intraoperative neurophysiological monitoring of motor functions and postoperative neurological deterioration, there was a change of the intraoperative motor signals in one patient who postoperatively showed an ischemic lesion in the right corona radiata. The intraoperative finding was an increase in the motor threshold for the left hand, but no change of motor signals for the face was noted. A left facial palsy with a quick improvement was noted postoperatively, but the left hand was intact. In the other 11 patients, there was no intraoperative change of motor signals*.* Table 2 describes the 18 patients with intraoperative monitoring and postoperative neurological deterioration.

**Table 2:** Description of the 18 patients with intraoperative monitoring and postoperative neurological deterioration

Nr	Asleep-awake surgery	Direct or delayed deterioration	Postoperative neurologic deficit	Complete regression	Probably cause	IOM change
1	Asleep	Direct	Hemiparesis	Almost	Resection of SMA	No
2	Asleep	Direct	Hemiparesis	Almost	Resection of SMA	No
3	Asleep	Direct	Facial palsy	Yes	Resection close to motor cortical area	Not from face decreased from hand
4	Asleep	Direct	Dysphasia and facial palsy	Yes	Resection close to/maybe of eloquent area + good plasticity postop	No, but did not record motor signals from face
5	Asleep	Direct	Hemiparesis	Yes	Resection close to motor areas	No
6	Asleep	Direct	Hemiparesis	Yes	Resection close to motor areas	No
7	Asleep	Direct	Paresis one arm	Yes	Resection close to motor areas	No
8	Asleep	Direct	Sensory deficits	Yes	Resection of eloquent areas + good plasticity postop	Sensory signals not recorded
9	Asleep	Direct	Sensory deficits	No	Resection of eloquent areas	Sensory signals not recorded
10	Asleep	Direct	Hemiparesis	Almost	Resection close to motor areas	No
11	Asleep	Delayed	Hemiparesis	Yes	Seizures	No
12	Asleep	Delayed	Hemiparesis + dysphasia	Yes	Seizures	No
13	Awake	Direct	Dysarthria, motor dysfunction of the tongue	Yes	Resection close (2 mm) to motor areas	Clinical: dysarthria, motor dysfunction of the tongue; motor signals intact.
14	Awake	Direct	Dysphasia	Yes	Resection close to speech areas	Stimulation induced dysphasia but no permanent changes
15	Awake	Direct	Verbal apraxia	Yes	Resection close to speech areas	Fluctuating stimulation induced verbal apraxia
16	Awake	Direct	Dysphasia	Yes	Resection close to speech areas	Fluctuating dysphasia, intraop evaluation of speech difficult
17	Awake	Direct	Dysphasia	Remained	Resection of eloquent tissue	Fluctuating dysphasia, intraop evaluation of speech difficult
18	Awake	Delayed	Dysphasia	Yes	Seizures + ischemia	Stimulation induced dysphasia but no permanent changes

*Nr* number, *IOM* intraoperative monitoring, *SMA* supplementary motor area, *intraop* intraoperative

**Fig. 2:**
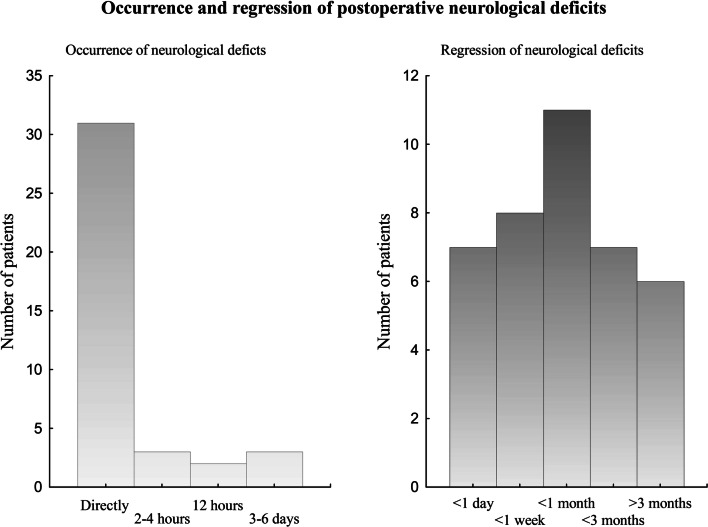
Occurrence and regression of postoperative neurological deficits in 41 patients. The left figure shows at what time after surgery neurological deficits occurred, and the right figure shows the time to regression of postoperative neurological deficits. No info = no information

The time course of the neurological deficits is shown in Fig. 2. In 32/41 patients (78%), the deficits occurred or worsened directly after surgery and in 9/41 patients (22%) after a delay, median (IQR) 12 (4–50) hours. In 27/41 patients (66%, 27% of the whole group of patients), there were complete regression of the new postoperative deficits. Six patients (6% of the whole group of patients) showed almost complete regression with only slight deficits remaining. In 6 patients (6% of the whole group of patients), the deficits still remained 3 months postoperatively, and for two patients, there was no information. The six patients with remaining neurological deficits are described in Table 3. In summary, all patients showed preoperative neurological deficits and had high-grade glioma (grades III–IV), mostly located in eloquent areas. There was no ischemic lesion in any of these patients. The probable reason for the neurological deficits was resection of eloquent tissue. In addition, two patients showed very fast tumour growth which probably contributed to an impaired plasticity and remaining deficits.

**Table 3:** Clinical characteristics of the patients with remaining neurological deficits

Preop deficit	Postop deficit	Diagnosis	Tumour location	Intraop finding	Grade of resection (%)	Postop ischemia	Probable cause deficits
Hemiparesis slight	Hemiparesis increased	Astro III	Frontal premotor	No IOM	84	No	Resection of eloquent tissue
Hemiparesis slight	Hemiparesis increased	GBM IV	Frontal motor	No signal changes	89	No	Resection of eloquent tissue in combination with very fast tumour growth
Ataxia	Paresis arm	GBM IV	Frontal motor	No signal changes	49	No	Resection of eloquent tissue in combination with very fast tumour growth
Hemiparesis slight	Cognitive	GBM IV	Frontal, CC	No IOM	96	No	Extensive tumour growth, resection of CC, old patient with no marginal
Sensory deficits	Sensory deficits increased	GBM IV	Parietal sensor	No motor signal changes	88	No	Eloquent tumour location and resection of eloquent tissue
Reading difficulties, cognitive deficits	Dysphasia	Astro III	Temporal, insular, dominant	Awake surgery. fluctuating dysphasia, no permanent changes	53	No	Eloquent tumour location and resection of eloquent tissue

*Preop* preoperative, *postop* postoperative, *intraop* intraoperative, *Astro III* astrocytoma WHO grade III, *IOM* intraoperative monitoring, *GBM IV* glioblastoma WHO grade IV, *CC* corpus callosum

In a subgroup analysis, we compared low-grade gliomas (WHO grade II), *n* = 24, with high-grade (WHO grades III–IV) gliomas, *n* = 69. We found that 12/24 (50%) patients with low-grade gliomas developed postoperative neurological deficits, 7 eloquent and 13 non-eloquent tumour locations, but there were complete or almost complete regression of neurological deficits in all these patients and no patients with low-grade gliomas showed remaining deficits. Among patients with high-grade gliomas, there were 27/69 (39%) patients with postoperative neurologic deterioration, 14 patients with eloquent tumour location and 13 patients with tumours in non-eloquent areas. Remaining deficits were found in 36% (5/14) of patients with eloquent tumour locations and in 8% (1/13) of patients with non-eloquent tumour locations. Thus, some trends were found with better recovery for patients with low-grade gliomas compared with high-grade gliomas (*p* = 0.15) and in the high-grade glioma group better recovery for patients with non-eloquent tumour locations compared with those with eloquent tumour locations (*p* = 0.16).

The two patients with a tumour in the supplementary motor area showed a postoperative hemiparesis with slightly slower movements (almost complete regression) after 3 months.

The four patients with a decline of a preoperative neurological deficit are included in the numbers above. A complete regression in those patients was defined as that the postoperative decline of the function returned to preoperative level.

The improvement occurred within one day, *n* = 7 (17%); within 1 week *n* = 8 (24%); within 1 month, *n* = 11 (27%); within 3 months, *n* = 7 (17%). For two patients there was no information after discharge from hospital.

**Fig. 3:**
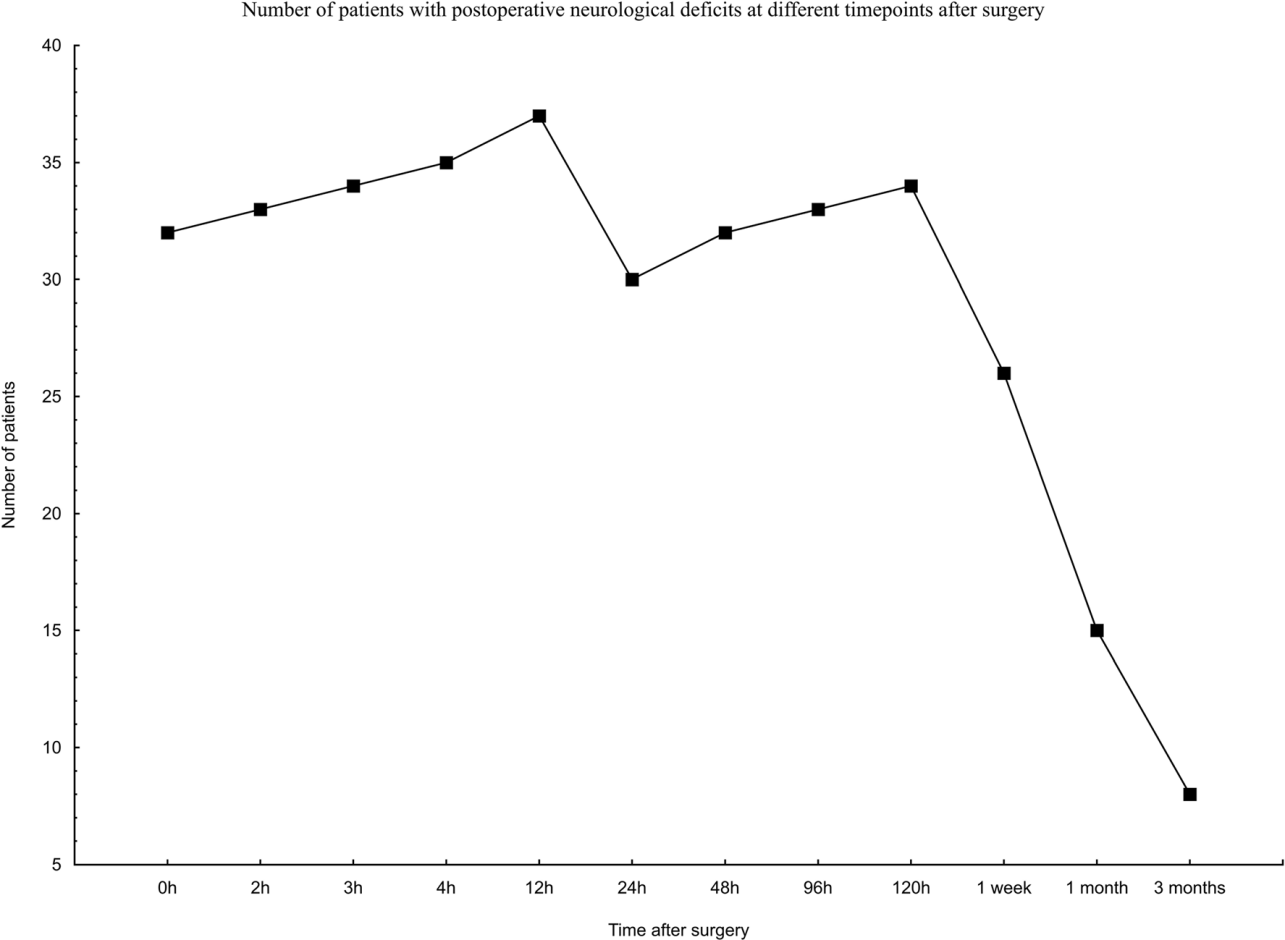
The number of patients with neurological deficits at different time points after surgery

Figure 3 shows the number of patients with neurological deficits at different time points after surgery.

### Seizures

EEG-verified seizures were detected in nine patients after surgery (seven patients < 24 hours and two patients > 24 hours after surgery) and caused postoperative deterioration in eight of these patients. In one patient with subclinical seizure activity for totally 22 h, no postoperative neurological deterioration was detected. In two other patients, there was a clinical suspicion that seizures caused a transient neurological deterioration, see below.

The result of the EEG and video recording postoperatively is published before [13].

#### Delayed postoperative neurological deterioration and seizures

In the nine patients with a delayed deterioration of the neurological function, seizures were the proven or the probable cause. The deterioration occurred after 2 h, 3 h, 4 h, 12 h (*n* = 2), 36 h, 50 h, 3 days and 6 days postoperatively. In seven of these patients, there were EEG-verified seizures that accounted for the deterioration. In one patient, the neurological deterioration occurred after the EEG monitoring had finished, but the patients displayed focal seizures that were considered the cause of the deterioration. In another patient with an aggravated paresis in one leg after focal seizures 12 h postoperatively, no epileptic seizure activity could be recorded although the clinical picture even in this case favoured seizures as contributing factor to the worsening of the deficits.

### Other complications:

In 14 patients (14%), postoperative MRI showed a new ischemic lesion and nine of them (64%) deteriorated neurologically after surgery. The ischemic lesion was considered a possible cause of the postoperative neurological deterioration in 5 cases, and in another patient, ischemia in combination with seizures was considered the probable cause.

In 4 of the 10 patients with postoperative ischemic lesions, intraoperative neurophysiological monitoring was used. There was a change of the intraoperative signals in one patient, described above. Table 4 describes the clinical characteristics of the patients with new postoperative ischemic lesion.

**Table 4:** Clinical characteristics of the patients with a new postoperative ischemic lesion

Number	Preop deficit	Postop deficit	Tumor location	Ischemia location	White matter bundles affected by ischemia	Ischemia caused deficits	Volume ischemia (cm^3^)	IOM perf/signal changes	When symptoms occurred postop	When regression symptoms	Grade of regression deficits	Postop seizures	Diagnosis
1	No	Cognitive	Bifrontal CC	CC, G Cing, N caudatus R	Cingulum, ATR	Yes	6.5	No	Directly	< 1 month	Complete		Pleomorft xantoastrocytom III
2	No	Hemipar	Front, G Cing L	G Cing L	CST, cingulum, CC, STR	Yes	4.4	Yes/no	Directly	< 3 months	Almost complete		GBM IV
3	No	Facial paresis	Temp R	Cor radiata, R	STR, CST, AF	Yes	3.3	Yes/yes	Directly	< 24 h	Complete		Oligo III
4	No	Dysphasia	Hippocamp L	Hippocamp L	ILF, Fo	No	0.2	No	Directly	< 24 h	Complete		GBM IV
5	No	Hemipar	Front, temp R	Inf front Gyr R	Cortico-striatal, ATR, cingulum	No	12	No	Directly	< 1 month	Complete		Astro II
6	No	Dysphasia	Front, temp, insula L	N caudatus, putamen, cor radiata, R	Anterior IC, cortico-striatal	Yes	0.2	Yes/no	50 h	< 1 month	Complete	Focal + generalized	Oligo II
7	No	Hemipar + cognitive	Front, temp, insula R	N caudatus, putamen R	AF, CST, STR, cortico-striatal, FAT	Yes	17	Yes/no	Directly	< 1 week	Complete		Astro III
8	Cognitive	Hemipar	Temp R	Temp R	ILF, vSLF	No	8.7	No	3 h	< 3 months	Complete	Focal + generalized	GBM IV
9	Dysphasia cognitive	Hemipar	Front, temp, insula, central L	Temp, cor radiata, L	ILF, Fo, IFOF, CST, STR	Yes	4.1	No	Directly	< 1 month	Complete		
10	No	No	Front L	CC, L	Genu CC		0.2	No	No symptoms	-	-	No	Oligo III
11	No	No	Front, G Cing R	CC, R	Splen CC, cingulum		0.3	No	No symptoms	-	-	No	GBM IV
12	No	No	Occ, temp R	Occ, R	ILF, OR, PTR, splen CC, MLF, IFOF, VOF		29	No	No symptoms	-	-		GBM IV
13	Visual field	No	Occ, temp L	Occ L	ILF, OR, PTR, splen CC, IFOF, cingulum		10	No	No symptoms	-	-		GBM IV
14	Visual field	No	Occ R	Occ R	ILF, OR, splen CC		7.1	No	No symptoms	-	-	Non-convulsive	Metastasis adeno-carccinom

*Nr* number, *Preop* preoperative, *Postop* postoperative, *IOM perf* intraoperative monitoring performed, *CC* corpus callosum, *G Cing* gyrus cinguli, *N* nucleus, *ATR* anterior thalamic radiation, *CST* corticospinal tract, *STR* superior thalamic radiation, *R* right, *Hemipar* hemiparesis, *Front* frontal, *L* left, *GBM IV* glioblastoma WHO grade IV, *Temp* temporal, *Cor* corona, *AF* arcuate fascicle, *Oligo II/III* oligodendroglioma WHO grade II/III, *Hippocamp* hippocampus, *ILF* inferior longitudinal fasciculus, *Fo* fornix, *Astro II/III* astrocytoma WHO grade II/III, *IC* internal capsule, *FAT* frontal aslant tract, *vSLF* vertical superior longitudinal fasciculus, *IFOF* inferior fronto occipital fasciculus, *Splen* splenium, *Occ* occipital, *OR* optic radiation, *PTR* posterior thalamic radiation, *MLF* middle longitudinal fasciculus, *VOF* vertical occipital fasciculus

Three patients underwent a second surgery after the primary tumour resection: One patient showed a preoperative hemiparesis which was aggravated after surgery. An expanding haematoma in the surgical field was noted and was evacuated 24 h postoperatively. After the second surgery, the patient improved to the preoperative level. Another patient was reintubated directly after the primary surgery due to decreased level of consciousness and received an intraparenchymal pressure monitoring device. The third patient displayed a generalized seizure and decreased level of consciousness. A CT scanning revealed a distant haematoma in the posterior fossa, and the patient was subjected to an external intraventricular drainage procedure.

### Summary of probable causes of postoperative neurological deterioration

To summarize, the probable causes of neurological deterioration in the 41 patients were EEG-verified seizures in seven patients, EEG-verified seizures + ischemia in one patient, clinical suspicion of seizures in two patients, resection of eloquent tissue in six patients, resection close to eloquent tissue in nine patients and resection of the SMA in two patients, ischemia in five patients (plus one described above with seizures) and postoperative haematoma in one patient. In eight patients, the reason for the transient deterioration was not clarified. We speculate that the probable reasons might have been a remaining effect of anaesthesia in two patients with a transient postoperative decline of a preoperative neurological deficit and multifactorial in four patients with a transient postoperative confusion. In two patients, no reasonable explanation could be found.

### Prediction of postoperative neurological deficits

In patients with a preoperative neurological deficit, a new postoperative neurological deficit developed in 21/40 (52.5%), compared with the patients with no preoperative neurological deficits, among whom 20/60 (33%) exhibited new postoperative neurological deficits, *p* = 0.06. Patients with tumours in presumed eloquent areas showed more new postoperative neurological deficits (22/39), 56% compared with patients with tumours in non-eloquent areas (19/61), 31%, *p* = 0.02. However, there was complete regression neurological deficit in 68% (15/22) of patients with tumours in eloquent areas which is comparable with patients with non-eloquent tumour location, 63% (12/19), *p* = 0.8. If complete and almost complete regression are included the numbers are 77% (17/22) and 84% (16/19) respectively, *p* = 0.7. The 2 patients lost to follow-up had tumours in non-eloquent areas and are not included in the calculations.

In patients with no neurological deficits postoperatively, a higher grade of resection was achieved, median (IQR) 100 (88–100) %, compared with patients with new neurological deficits postoperatively, 81 (60–99) %, *p* = 0.002. There was no difference in the distribution of tumour diagnoses between patients with and without new postoperative neurological deficits.

The parameters used in the simple regression analysis were age (continuous), sex (male/female), tumour grade (low/high/other), preoperative neurological deficits (yes/no), presumed eloquent tumour location (yes/no) and tumour volume (continuous). The results are shown in Table 5. The variables chosen from the simple regression analysis to be examined in the multiple regression analysis were preoperative neurological deficits (*p* = 0.057) and presumed eloquent tumour location (*p* = 0.012) together with age and sex. In the multiple analysis did a presumed eloquent tumour location become a significant predictor of postoperative neurological deterioration *p* = 0.027 (see Table 5).

**Table 5:** Simple and multiple regression analysis: risk factors for postoperative neurological deficits and for remaining neurological deficits. The upper part shows the results of the simple and multiple regression analysis regarding possible risk factors for postoperative neurological deficits including correction for age and sex for the 100 patients in the study. The lower part of the table shows the result if the simple regression analysis of possible risk factors for remaining neurological deficits among the 39 patients with new postoperative neurological deficits and available follow-up data. Factors with a *p* value < 0.1 in the simple regression analysis were chosen to be tested in the multiple regression analysis and a *p* value < 0.5 was considered statistical significant

	Univariate analysis		Multivariate analysis		Multivariate analysis with correction for age and sex	
	OR (95% CI)	*p*	OR (95% CI)	*p*	OR (95% CI)	*p*
Parameters of new postoperative neurological deficits (*n* = 100)						
Age	0.95 (0.76–1.2)	0.60	-	-	0.95 (0.76–1.2)	0.60
Sex	1.1 (0.87–1.3)	0.57	-	-	1.0 (0.86–1.3)	0.68
Tumour grade	0.93 (0.76–1.1)	0.50	-	-		
Preop neurological deficits	1.2 (0.99–1.5)	0.057	1.2 (0.9–1.4)	0.16	1.1 (0.90–1.4)	0.31
Presumed eloquent tumour location	1.3 (1.0–1.6)	0.012	1.2 (1.0–1.5)	0.031	1.3 (1.0–1.5)	0.027*
Tumour volume	0.86 (0.71–1.0)	0.14	-	-	-	-
Parameters remaining postoperative neurological deficits (*n* = 39)						
Age	0.89 (0.63–1.2)	0.48			1.0 (0.72–1.4)	0.88
Sex	0.88 (0.63–1.2)	0.45			0.88 (0.63–1.2)	0.45
Tumour grade	0.75 (0.55–1.04)	0.08	0.50 (0.64–1.4)	0.71	0.96 (0.65–1.4)	0.83
Preop neurological deficits	1.5 (1.1–2.1)	0.009	1.5 (1.0–2.1)	0.049	1.5 (1.0–2.2)	0.046*
Presumed eloquent tumour location	1.3 (0.91–1.7)	0.16				
Preoperative tumour volume	1.1 (0.81–1.6)	0.49				
Timepoint for deficits in relation to surgery	1.1 (0.79–1.5)	0.59				
Postoperative ischemic lesion on MRI	1.3 (0.91–1.8)	0.15				

*Significant in the multivariate analysis

### Prediction of remaining neurological deficits

For calculating risk factors of remaining neurological deficits, the following parameters were used: age, sex, tumour grade (high/low/other), preoperative neurological deficits, presumed eloquent tumour location, preoperative tumour volume, when the postoperative neurological deficits occurred in relation to surgery (continuous) and postoperative ischemic lesion on MRI (yes/no). The variables chosen to be examined in the multiple regression analysis were tumour grade (*p* = 0.08) and preoperative neurological deficits (*p* = 0.009) together with age and sex. In the multivariate analysis, preoperative neurological deficits became a significant predictor of remaining neurological deficits, *p* = 0.046 (see Table 5).

## Discussion

To summarize this study, postoperative neurologic deterioration occurred in 41% of the patients, and patients with tumours in presumed eloquent areas showed more often new postoperative neurological deficits compared with patients with tumours in non-eloquent areas. The probable cause of postoperative neurologic deterioration was EEG-verified seizures in seven patients, a new ischemic lesion in five patients, both of these in one patient and postoperative haematoma in one patient. In 11 patients, tumour resection close to eloquent areas including the SMA was considered the probable cause of neurologic deterioration, and in six patients, the resection included eloquent tissue resulting in neurological deficits after surgery. In the majority of patients (78%), the deficits occurred directly after surgery, and in the nine patients with a delayed neurological deterioration, seizures were the proven (*n* = 5) or probable (*n* = 4) cause of the new deficits. In 66% of the patients with postoperative deficit (27% of the whole group), there was complete regression of the postoperative deficits, and in another 15% of the patients with postoperative deficit (6% of the whole group), there was almost complete regression with a slight disturbance of the function remaining after 3 months. Remaining deficits were found in 6% of all patients, and all these patients showed preoperative neurological deficits and high-grade tumours with mainly eloquent locations. Eloquent tumour location became a predictor of postoperative neurological deterioration, and preoperative neurological deficits were a predictor that the deficits would remain.

### Neurological deterioration

The incidence of any neurological deterioration after craniotomy for primary brain tumours in our study was 41%, which is higher than previously described [6, 7, 21, 15, 14, 27, 22, 32]. However, the decline in the neurological function was in the majority of patients transient, with complete or almost complete regression of the symptoms in 81% of the patients and a complete regression in 66% of the patients. Thus, the incidence of permanent postoperative neurological deficits was lower, 6%, in the whole group of patients, and another 6% of the patients reported a slight remnant deficit which did not impair function. These numbers are comparable with the incidence, 7–20% [14, 15, 21, 27, 4, 39, 38] of postoperative neurological deficits, described earlier. A meta-analysis of outcome in glioma surgery showed that early deficits occurred in 30% of patients [9], and in a study by Gempt et al., transient postoperative neurological deficit was found in 17% of newly diagnosed, and 32% of recurrent gliomas and permanent neurological deficits were found in 7 and 16% respectively [15]. Sawaya et al. [27] described neurological complications in 8.5%, Lonjaret et al. [21] in 16% and Brell et al. [4] in 20.5% of patients after surgery for brain tumours. In a study by Berger et al., immediate motor deficits were found in 22% and speech deficits in 3% of patients [3].

Our study shows that the deficits occurred directly after surgery in 78% of the patients who developed deficits, which is in line with previous studies [21]. In Lonjaret’s study, 85% of the patients showed neurologic complication during the first 2 h after surgery [21], and the central nervous system was the dominating location for postoperative complications within the first 24 h after brain tumour surgery [37].

The reason for neurological deterioration may be direct tissue damage after surgical manipulation or an effect of resection of eloquent tissue. Neurological deterioration may also occur secondary to tissue oedema, arterial ischemia, venous infarctions [15, 16], vasospasm after vessel tears [23], haematomas [16] or be an epileptic ictal or postictal phenomena. Symptoms due to surgical manipulation, resection or ischemia are expected to occur immediately after surgery, whereas postoperative haematomas, epileptic ictal phenomena and symptoms due to venous infarctions or vasospasm may occur after a delay [16]. A transient neurological deterioration could also be due to unmasking of an already existing deficit or borderline function which could be compensated for under fully alert and awake conditions but is revealed after anaesthesiology. In this study, 6% of the patients showed a new ischemic lesion on MRI as a plausible cause of the postoperative neurological deficits and more patients with tumours in presumed eloquent areas developed new postoperative neurological deficits. Two patients developed a hemiparesis with almost complete regression after surgery in the supplementary motor area, which is a well-known phenomenon after surgery in this area [17]. Plasticity due to reorganisation of the function is a plausible explanation of the improved function after a certain period of time in those cases [12].

In a previous work [13], we examined the occurrences of epileptic seizures with continuous EEG monitoring after surgery for presumed primary brain tumours. We found that 7% of the patients displayed postoperative epileptic seizures the first 24 h after surgery [13]. However, in all 9 patients (9%) in this study with a delayed neurological deterioration, seizures were the proven (*n* = 5) or probable (*n* = 4) cause of the new deficits.

In six patients (6%), there were remaining deficits after 3 months. These patients all showed preoperative neurological deficits and harboured tumours in or in close connection to the motor, sensor and language areas, and the diagnosis in all patients was high-grade gliomas which showed a very fast regrowth in at least two of them. The perioperative neurophysiological monitoring used in 3 of these patients did not show any warning signs of impaired motor function. Thus, the monitored motor function seemed to be neurophysiologically intact, and the plasticity and capacity of improvement may have been impaired by the aggressive growth of their high-grade tumours. When we compared the recovery of postoperative neurological deficits between low- and high-grade gliomas, we found some trends with a better recovery in the group of patients with low-grade gliomas, in which no patients showed remaining deficits. This should be compared with the group of patients with high-grade gliomas in which 6/27 (22%) of patients showed remaining deficits and 5/6 (83%) of these patients had tumours in eloquent areas. These numbers are too small for statistic calculations, but we think this trend seems to be reasonable and favours the fact that patients with low-grade gliomas have a better plasticity due to the slow growth of the tumour.

The substantial variation of the incidence of postoperative neurological deterioration after brain tumour surgery found in the literature could be explained by the differences in the methodology of the studies, heterogenicity of the materials, i.e. if tumours in eloquent areas are included [16] and methods of detecting the complications [10] and definitions of neurological complications. In a prospective study, with the goal of reporting the incidence of neurological complications, a higher incidence is to be expected due to more meticulous schedules for detecting any decline of neurological deterioration compared with a retrospective analysis based on register data. Our study provides data on a detailed level of value for increasing the knowledge of short-term surgical outcome. This kind of data is of value for the preoperative information to the patients and could be helpful in the decision process regarding the indication for surgery when weighing possible benefits and risks and for optimizing the perioperative treatment of the individual patient [29].

### Postoperative ischemic lesions

Our numbers of postoperative ischemic lesions (14%) are lower than in the study by Gempt et al. [15], who identified new postoperative ischemic lesions in 31% of newly diagnosed and in 80% of patients with recurrent gliomas. Other have described new ischemic lesions after glioma surgery in 23% [3], 64% [30] and 70% [34] of patients. Tumour location in proximity to perforating arteries [15], insular tumours [3] and recurrent gliomas [3, 15] has been identified as a risk factor for postoperative ischemic lesions, and age was found to be an independent risk factor for stroke within 30 days after brain tumour surgery [2]. Vascular reorganization or vessel obliteration by brain irradiation was suggested as an explanation for the increased number of ischemic lesions after resection of recurrent gliomas [15]. A higher probability of new postoperative neurological deficit has been identified in patients with a new postoperative ischemic lesion [15]. In our study, there was no significant difference of the occurrence of neurological deficits in patients with or without ischemic postoperative lesions on MRI. None of the patients with remaining neurologic deficits showed ischemic postoperative lesions, and new postoperative ischemic lesions could neither be identified as a predictor of remaining neurological deficits in this small group of patients.

### Postoperative haematoma

The incidence of haematomas requiring evacuation in this study was 1%. Even if the reported incidence of postoperative haematoma after craniotomy varies a lot [28], this is in line with previous studies [21, 37, 18, 43].

### Risk factors for neurological deterioration

We found that more patients with preoperative neurological deficits developed new postoperative neurological deficits compared with the patients with no preoperative neurological deficits preoperatively with border significance (*p* = 0.06), but preoperative neurological deficits did not become an independent predictor of postoperative neurologic deterioration. However, this finding is in accordance with our clinical experience, and it is possible that preoperative neurological deficits would have turned out to be a predictor for postoperative new neurological deficits in larger a group of patients. In patients with preoperative neurological deficits, there was a higher probability that the postoperative neurological deterioration would remain, at least more than 3 months. Preoperative neurological deficit, as a risk factor for remaining postoperative neurological decline, has also been recognised by others [32, 14] and altered mental status at presentation and tumour-related neurological deficits are independent risk factors for postoperative mortality in brain tumour patients [2]. Preoperative neurological deficits are also recognized as a risk factor for postoperative complications in general [25, 2]. However, this is in contrast to Lonjaret et al.’s finding that the absence of a preoperative motor deficit was significantly associated with a neurologic complication [21].

A postoperative decline in the neurological function is expected if the tumour is located in eloquent areas, and the resection is performed close to the cortical or subcortical areas harbouring the function. We found that patients with tumours in presumed eloquent areas more often developed new postoperative neurological deficits compared with patients with tumours in non-eloquent areas, and eloquent tumour location was an independent predictor of postoperative neurological deterioration. The postoperative neurological deficits in patients with presumed eloquent tumour location showed a high tendency (71%) of complete regression. This can be expected, since neurophysiological intraoperative monitoring of cortical and subcortical motor functions, in some cases combined with awake surgery for monitoring of speech functions and visual fields, is used if the tumour is located adjacent to these eloquent areas. Except for one patient, there was no change of relevant intraoperative motor signals in those patients who developed postoperative motor deficits after surgery, and the high incidence of fast and complete regression of the deficits, at least in low-grade tumours, favours that the function was disturbed by the close resection, and the areas harbouring the function was actually intact. This result is in line with our experience of patients with low-grade gliomas and intact intraoperative motor potentials as a tool to predict complete recovery of postoperative motor deficits, i.e. in case of intact intraoperative neurophysiological monitoring of motor potentials and postoperative motor deficits, the motor function will usually recover completely. However, our study indicates that there might be a difference between low- and high-grade tumours in this respect. In low-grade tumours, there were no remaining deficits, irrespectively if the tumour was located in eloquent or non-eloquent areas. But in high-grade tumours, more patients with eloquent tumour locations showed remaining deficits, and in two patients, there were remaining motor deficits, although intraoperative motor signals did not change during surgery. Thus, even if our numbers are small and we cannot draw any safe conclusions, our results indicate that we might be extra careful in a patient with preoperative neurological deficits and a suspicion of high-grade tumours who may have an increased risk of remaining new postoperative neurological deficits. Also, motor potentials from the face area could be more difficult to receive adequately, which we experienced with one patient with a postoperative facial paresis.

In our group of patients, a significant higher grade of resection was achieved in patients with no neurological deficits. Our interpretation of this finding is that a less grade of resection correlates with and represents eloquent and often widespread tumour growth which makes the tumour unresectable. Since there is a probable correlation between the grade of resection and eloquent tumour location, the latter was chosen to be used in the analysis of possible risk factors for postoperative neurological deterioration and remaining deficits.

A similar finding was made by Fadul et al. (1988), who already showed that patients with complete resection had fewer neurologic complications compared with patients with biopsy or less extensive procedures [14] which also is an observation by others [27, 22, 36].

As discussed above, awake surgery with intraoperative neurophysiological monitoring is used in order to maximise the grade of resection without causing postoperative neurological deficits [11, 26] in cases with tumour locations harbouring eloquent functions that are possible to monitor intraoperatively. With regard to the findings that the majority of neurological deficits are transient, one may speculate that that some of the neurological deterioration during awake surgery causing a termination of the surgery would end in recover, and it is possible that in some cases, especially low-grade tumours with higher plasticity, more tumours could have been removed without permanent neurological deficits.

Other factors identified as important for the neurological outcome of the patient after surgery is the experience of the surgeon and the volume of surgeries performed in the surgical centre [1, 24, 33]. Regarding reoperations, there are both reports with elevated risk for complications [6] and no increased risk [8, 14] after a secondary craniotomy for brain tumours.

### Shortcomings of the study

One shortcoming is that the number of the patients is quite small, and for calculating risk factors, a larger number of patients would have been preferred. Also, we did not use an established motor scale, but the evaluation of the motor deficits was done according to our motor scale used in the clinical practice. However, the goal was to find out if the deficits were transient or not, and we think that the scale used served this purpose. The advantage of the study is that the consecutively included patients are individually evaluated on a detailed level in the acute phase after surgery. In two patients, information regarding the postoperative course after discharge from hospital was lacking.

## Conclusions

After surgery for primary brain tumours, neurological deterioration occurred in 41% and remaining deficits in 6% of patients. Only patients with preoperative neurological deficits and high-grade tumours mainly in eloquent areas showed persistent deficits. Epileptic seizures accounted for the majority of delayed neurological deterioration. Eloquent tumour location was a predictor of postoperative neurological deterioration, and the presence of preoperative neurological deficits was a predictor of remaining new postoperative neurological deficits.

## Electronic supplementary material

ESM 1 (JPG 1.75 mb)
