# Supplementary figures and images for: Time course of neurological deficits after surgery for primary brain tumours
Source: Acta Neurochir (Wien). 2020 Jul 2;162(12):3005–18. doi: 10.1007/s00701-020-04425-3 (PMC7593278; doi:10.1007/s00701-020-04425-3)

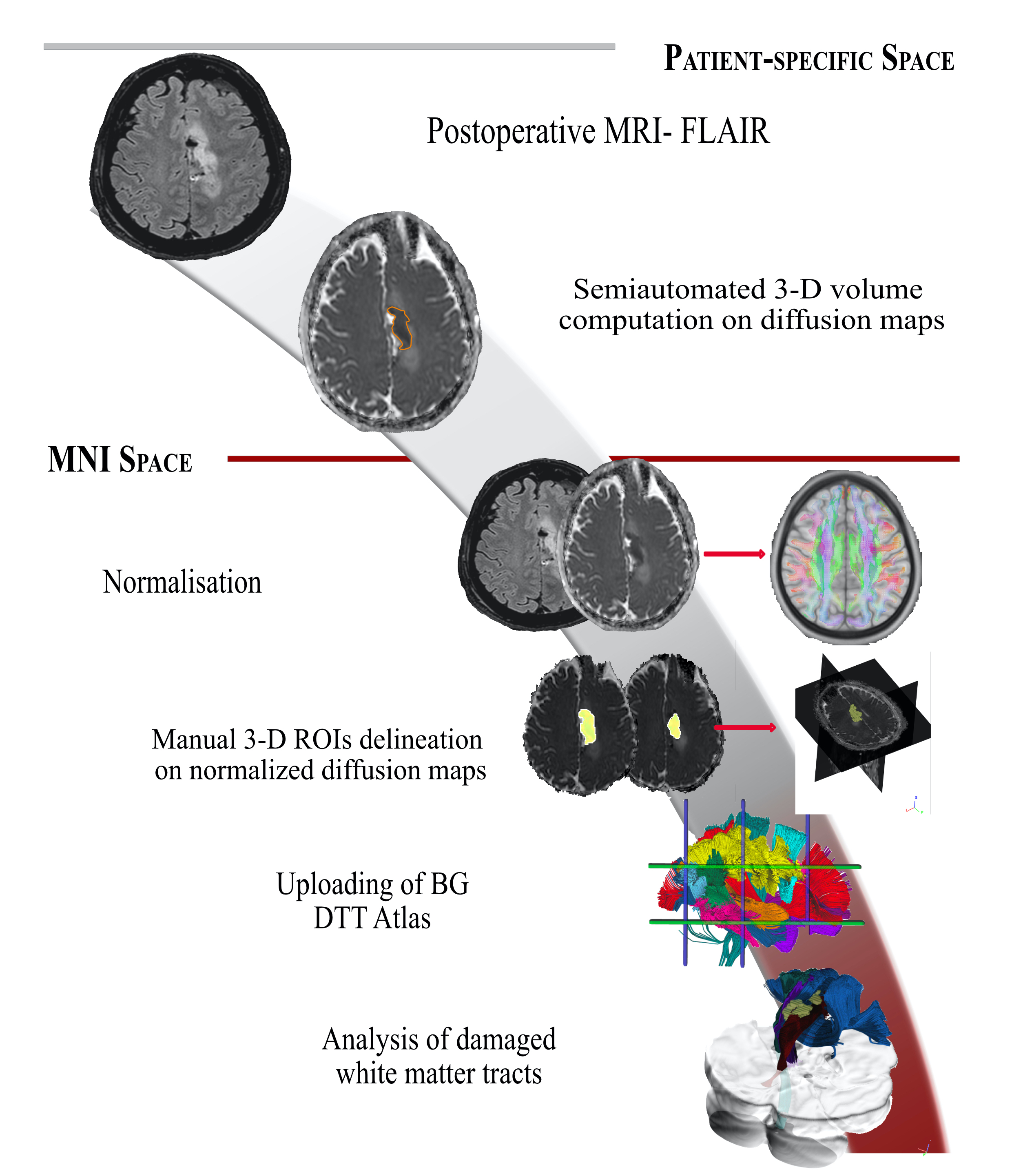

Supplement: Supplementary file 1 — (JPG 1.75 mb) [file 701_2020_4425_MOESM1_ESM.jpg]
